# Challenges and Opportunities in Preserving Key Structural Features of 3D-Printed Metal/Covalent Organic Framework

**DOI:** 10.1007/s40820-024-01373-w

**Published:** 2024-03-21

**Authors:** Ximeng Liu, Dan Zhao, John Wang

**Affiliations:** 1 Department of Materials Science and Engineering, National University of Singapore, Singapore, 117574 Singapore; 2 Department of Chemical and Biomolecular Engineering, National University of Singapore, Singapore, 117585 Singapore; 3 National University of Singapore (Chongqing) Research Institute, Chongqing, 401123 People’s Republic of China

**Keywords:** Metal–organic frameworks, Covalent organic frameworks, 3D printing, Microstructure, Monolith

## Abstract

Metal–organic framework (MOF) and covalent organic framework (COF) are a huge group of advanced porous materials exhibiting attractive and tunable microstructural features, such as large surface area, tunable pore size, and functional surfaces, which have significant values in various application areas. The emerging 3D printing technology further provides MOF and COFs (M/COFs) with higher designability of their macrostructure and demonstrates large achievements in their performance by shaping them into advanced 3D monoliths. However, the currently available 3D printing M/COFs strategy faces a major challenge of severe destruction of M/COFs’ microstructural features, both during and after 3D printing. It is envisioned that preserving the microstructure of M/COFs in the 3D-printed monolith will bring a great improvement to the related applications. In this overview, the 3D-printed M/COFs are categorized into M/COF-mixed monoliths and M/COF-covered monoliths. Their differences in the properties, applications, and current research states are discussed. The up-to-date advancements in paste/scaffold composition and printing/covering methods to preserve the superior M/COF microstructure during 3D printing are further discussed for the two types of 3D-printed M/COF. Throughout the analysis of the current states of 3D-printed M/COFs, the expected future research direction to achieve a highly preserved microstructure in the 3D monolith is proposed.

## Highlights

- A comprehensive investigation on the research states of 3D-printed metal/covalent organic frameworks (M/COFs) is conducted with the discussion on the M/COF-mixed monolith and M/COF-covered monolith separately.
- Recent advances in design strategies regarding both the paste/scaffold formation and the 3D-printing/covering process for preserving the better structural features of M/COFs (surface area, porosity, and micromorphology) in their 3D printed monolith are overviewed and discussed.

## Graphical abstract

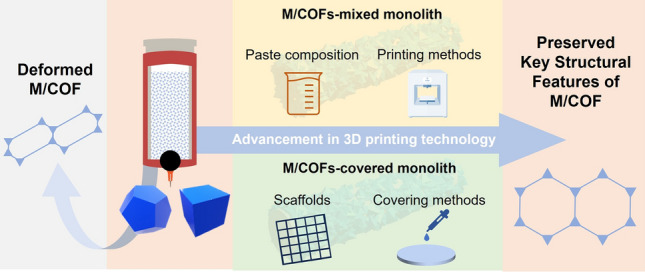

## Introduction

Metal–organic frameworks (MOFs) and covalent organic frameworks (COFs) are unique porous materials formed by periodical connections between individual building units. The special structure enables precise tailoring of the framework and functionality of MOFs and COFs through adjusting the building units to achieve targeted properties. MOFs contain both inorganic and organic components, which are termed as metal nodes and organic linkers, respectively. The linker will connect the metal nodes to form a framework structure, and the length of the linker has a great impact on the pore size and surface area. On the other hand, COFs are constructed only by the linkage between the long organic chains through covalent bonds, which makes them having larger pores than the MOFs. The MOFs and COFs (M/COFs) exhibit superior microstructural features, including adjustable pore size, abundant pore volume, and tremendous functionalization variability, which are required for a large range of applications. Due to their diverse structural features, M/COFs have found wide applications in various areas, e.g., gas storage/separation, sensing, liquid treatment, luminescence, energy storage/conversion, and biomedicine [1, 2]. Recently, more efforts have been made to promote the commercialization of M/COFs, such as the ION‐X^®^ gas storage and delivery system that utilizes MOFs as adsorbents [3]. One of the main limitations hindering their further commercialization is that M/COFs are typically fabricated in powder forms, which makes transportation, integration, and recycling challenging. Consequently, further cost reduction is impeded, thereby hindering large-scale production. Moreover, achieving high volumetric performance has become a top priority in many fields. The loosely packed M/COF powders containing a large amount of void space are difficult to achieve such goals. To address the aforementioned technical issues and advance industrialization in the near future, investigating the shaping of M/COF powders into a 3D architecture with preserved microstructure is a promising direction and presents large value for developing next-generation functional materials. Therefore, it is important and timely to review the state-of-the-arts 3D printing techniques which have limited negative effects on the microstructures for MOFs and COFs.

3D printing technology is considered one of the most promising methods for the fabrication of complex 3D architectures, which are otherwise impossible by conventional techniques [4, 5]. This technology can build up the 3D monoliths layer-by-layer with precise control of the pattern in each layer [6]. Compared to traditional shaping methods, such as molding, pelleting, or casting, 3D printing allows for the creation of complex architectures with a high designability and low material waste [3, 7]. Since 2014, the number of research studies on 3D-printed M/COFs has been continuously growing, which involves a wide range of applications and various M/COF materials. These investigations have demonstrated the promising performance achieved by the 3D-printed M/COF monoliths [6]. It is envisioned that the incorporation of 3D printing techniques and M/COF materials will lead to a bright future for shaping of the functional M/COF powders into advanced 3D solid structures [8].

**Fig. 1:**
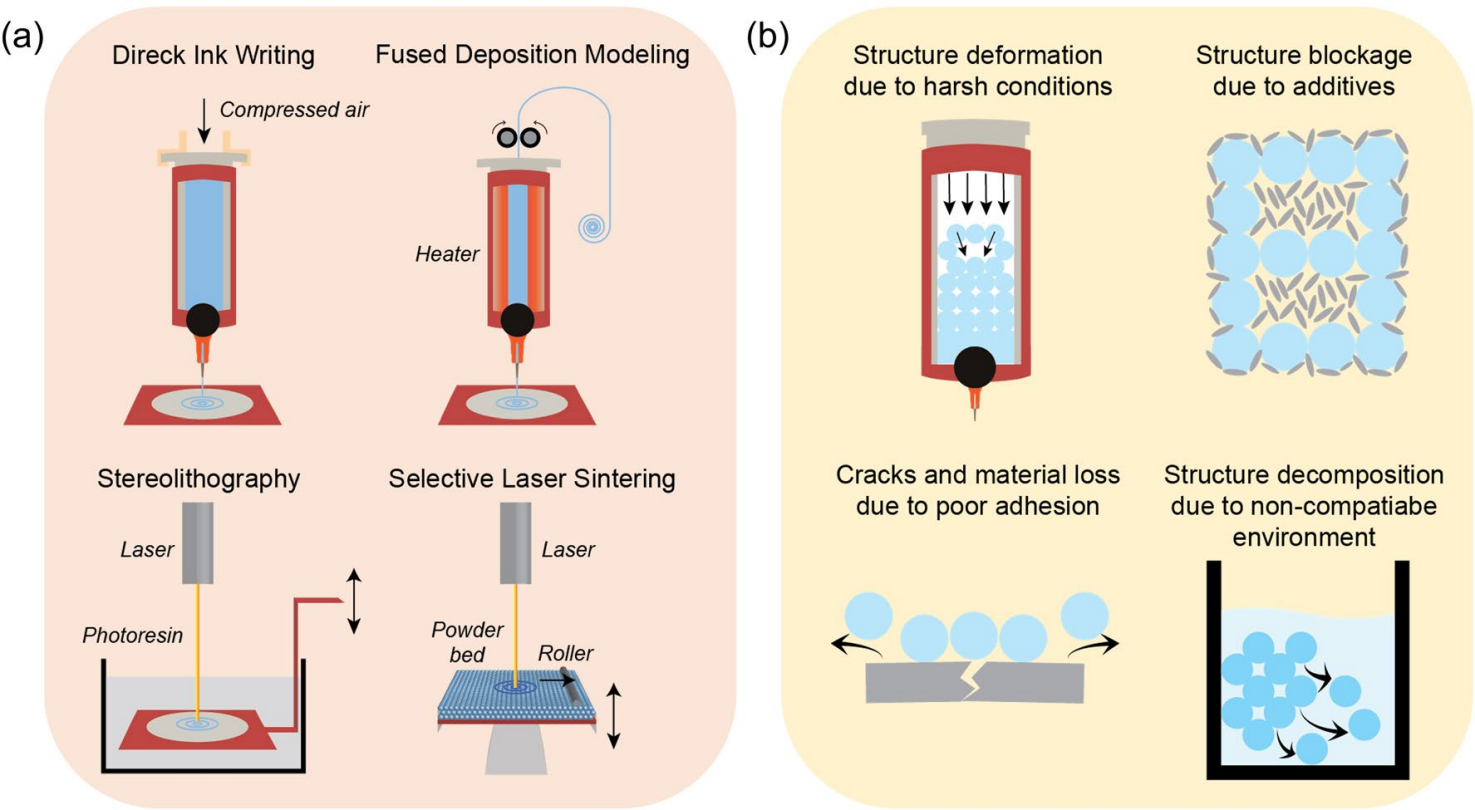
**a** Illustration of different 3D printing technologies. **b** Illustration of reasons contributing to the current challenges for 3D-printed M/COF monoliths

Comparing to other materials, M/COFs are more difficult to be 3D-printed. They have less suitable rheological properties to allow them being extruded smoothly and they are also hard to be entangled, cured, or sintered by themselves, making the 3D printed monolith easy to crack. Various 3D printing technologies have been developed for fabricating 3D-printed M/COFs and are illustrated in Fig. 1a. The most commonly used one is direct ink writing (DIW), in which active materials are mixed with additives and solvents to form a paste and are extruded through a fine nozzle to create a precise pattern. Due to its simplicity and lack of the need for high-temperature or other harsh requirements [9, 10], it is the most suitable 3D printing method for M/COFs to date. In DIW, the viscosity of the paste is a key parameter affecting the printability. During the printing, it requires a low viscosity at high shear rates for a continuous flow of the paste from the nozzle, and a high viscosity at low shear rate to prevent spreading of the ink after deposition on the plate [11, 12]. One study states if the viscosity is around 10 Pa·s at a shear rate of 0.2 s^−1^, the 3D structure cannot be maintained [13]. Other methods, such as fused deposition modeling (FDM), stereolithography (SLA), and selective laser sintering (SLS), are also employed for 3D printing M/COFs. In these printing processes, the active materials are mixed with thermoplastic binders or UV-curable resins. For FDM, the paste is first made into a filament, which is then extruded by melting and subsequently solidified by cooling down. For SLA and SLS, a laser is focused onto the polymer resin/powder to draw a pre-designed pattern, and a 3D monolith is then obtained through curing or sintering [8]. There are also other types of 3D printing technologies, such as digital light processing (DLP) which uses a beam of UV light to cure the polymer resin and form a whole layer at once [14]. However, since their mechanism is similar to the above ones, they will not be discussed in detail in this review. Those types of 3D printing have lower degree of requirements on viscosity and often does not require precise adjustment of the paste composition for an ideal rheological property. A 3D monolith can be easily obtained by these types of 3D printing technology, as long as the content of additives is high enough to maintain the shape after cooling or UV-curing. However, this can lead to a low M/COF content in the paste and, in-turn, a small surface area, such as the MOF-Nylon-12 monolith with a surface area of only 40 m^2^ g^−1^ [15]. In addition, more advanced 3D printing technologies have been developed, such as injection continuous liquid interface production, which allows for using more viscous paste and fast print speed [16], rotational multimaterial 3D printing, which can create helically architected filaments [17], multimaterial multinozzle 3D printing, which enables fast switching of materials during extrusion [18], 3D printing coupling with machine learning [19], and 4D printing which enables the materials to respond to external stimuli [20, 21]. Although these advanced technologies have not been applied in the M/COFs yet, they present a great potential for the future development on the 3D printed M/COFs.

The current challenge faced by 3D-printed M/COF monoliths is that they generally exhibit lower specific surface areas and pore volumes compared to their powder counterparts. Several reasons contributing to this problem are shown in Fig. 1b: (I) high temperature and pressure during the printing process cause M/COF crystals to deform or decompose; (II) additives in the paste can lead to pore blockage, surface covering, and accumulation of dead mass; (III) poor adhesion between the M/COFs and additives/skeleton can result in the 3D structure collapse and material loss; (IV) the solvent in the paste or the working environment can corrode the M/COF structure. Avoiding these issues and maintaining a high mass loading of the M/COF materials in the 3D monoliths without compromising their unique properties would benefit various applications [22]. Numerous efforts have thus been made to achieve facile printing condition, reduce additives, enhance interactions, and prevent M/COF degradation. There has been some excellent reviews on the topic of 3D printed MOFs or COFs. For example, Hussain et al. have thoroughly introduced the 3D printed MOFs and COFs and their related applications, respectively [23]. D’Alessandro et al. have also overviewed different 3D printing technologies and the applications of 3D printed MOF in the clean energy and environmental fields [24]. However, a comprehensive review for the specific topic about preserving key structural features of MOF and COF during the 3D printing is still lacking. Therefore, this review aims to bridge the gap and introduce the efforts made to preserve the desired microstructure features of M/COFs in 3D-printed M/COF monoliths, which are critically important for targeted applications. In the discussion, the promising fabrication strategies are categorized into the M/COF-mixed monolith and M/COF-covered monolith, as well as the paste and skeleton composition in these two distinct types of 3D-printed M/COFs. This overview is designed to fill in the large gaps and inspire new ideas to further advance 3D-printed M/COF monoliths. In some works, the M/COFs have been utilized as a filler instead of the active materials to enhance the properties of 3D-printed monoliths, such as improving mechanical strength [25], promoting polymer crosslinking [26], capturing electrolyte ions [27], storing drugs [28], and in some applications requiring only a small amount [29, 30], with loadings typically less than 3 wt%. Hence, those examples are not included in the discussion.

## Current States of 3D-Printed M/COF Monolith

The combination of both macro- and micro-structure in 3D printed M/COFs shows many extra advantanges as compared to their powder counterparts, which allows them delivering better performance in many applications. The most studied advantages is the acceleration of mass transport process due to the plenty of tunnel formed by the interconnected macrostructural monolith. It can help to improve the flow of gas or liquid and therefore enhance the ability of pollutant removal, breakthrough gas separation, etc. Banerjee et al. have demonstrated that the 3D printed COF monolith can remove 98% of methyl blue pollutant from the water within 30 s, while the powder counterparts can only absorb 73% MB after 5 min [31]. In addition, 3D printing assembles the powder particles into a bulk part with sufficient mechanical strength, which makes it easier to handle, transport, and recycle. When operating under high gas/liquid flow rate, the 3D printed monolith thus can be more stable and prevent the material loss, comparing to powder form. It has also been demonstrated that the 3D-printed monolith can help to guide the deposition of other species, such as sodium, lithium, or zinc, which is useful in the energy storage field to prevent the dendrite formation or accelerate the materials conversion [32].

**Fig. 2:**
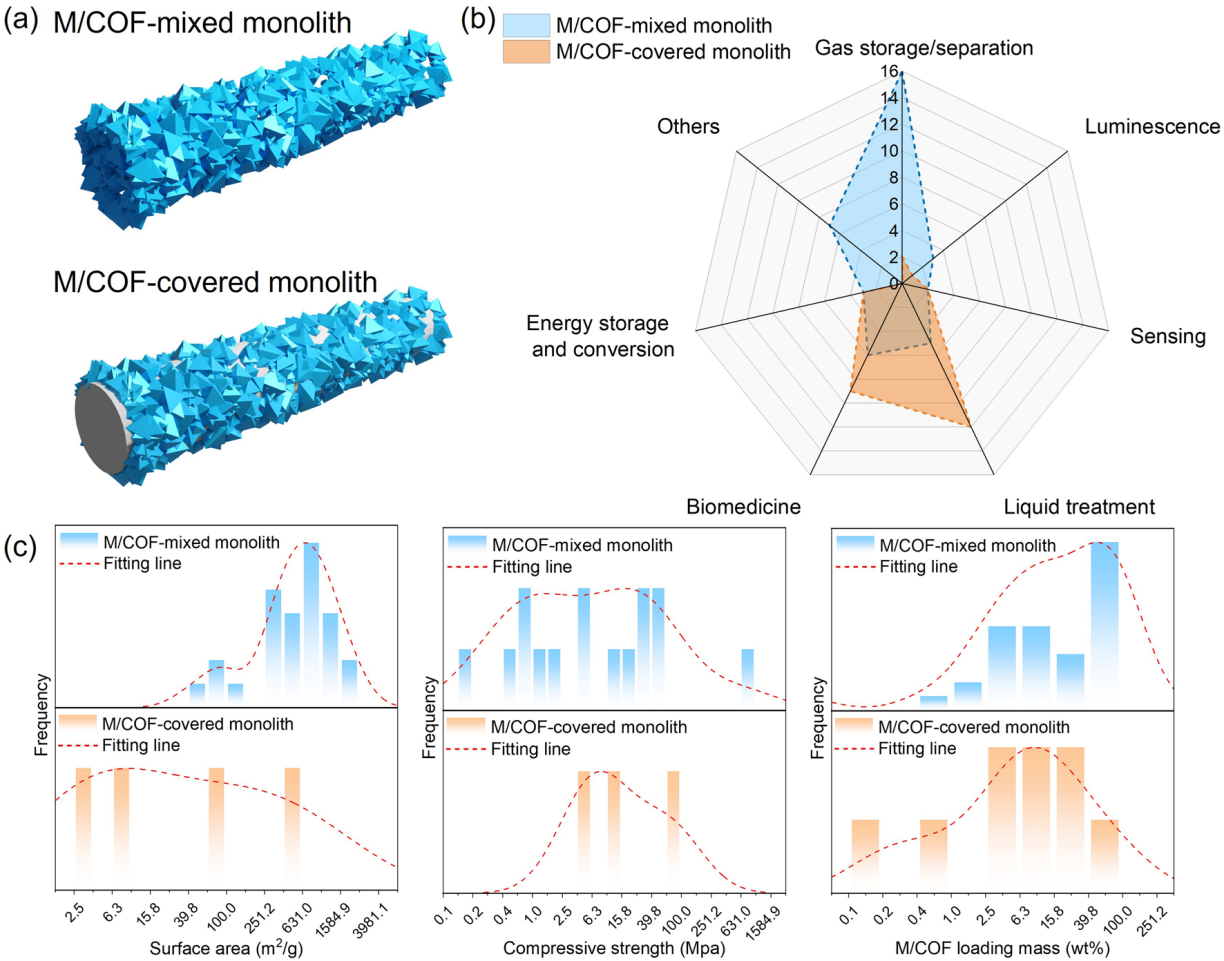
**a** Illustration of the two types of 3D-printed M/COF monolith. **b** Number of published works in terms of different applications. **c** Distribution of reported values on surface area, compressive strength, and M/COF loading mass in the published works about 3D-printed M/COF. The red dash line is the 5th degree polynomial fitting curve

An ideal 3D-printed M/COF monolith should combine both the advantages of 3D architecture and M/COF intrinsic structural properties. Therefore, it requires a strong mechanical strength to maintain the architecture with good scalability and reusability of the monolith, as well as retaining large surface area and porosity of the M/COF particles with inherent functionalities [33]. Typical 3D-printed M/COFs exist in two different forms, as shown in Fig. 2a. One form involves the M/COF-mixed with additives in the monolith, which is 3D-printed using a paste containing both M/COFs and additives/solvents. The other form includes the M/COF-covered on the surface of a 3D monolith, which is fabricated by coating the M/COFs on a pre-developed 3D-printed skeleton. Through the analysis of publications on 3D-printed M/COFs, it is suggested that the application fields for the two different types of 3D M/COF monoliths show distinct differences, as indicated in Fig. 2b. The M/COF-mixed monolith is generally used in gas storage/separation applications, while the M/COF-covered monolith is more commonly used in the biomedicine field, such as bone implant, and in liquid treatment, such as dye removal. The difference is mainly due to the unique properties of the two types of monoliths. Figure 2c shows the distribution of the reported values regarding to different mechanical properties, surface area, and mass loading for the 3D M/COF-mixed monolith or 3D M/COF-covered monolith. In general, the M/COF-mixed monoliths can provide higher M/COF loadings and surface area. Most reported M/COF loadings can reach over 40 wt%, and the surface area is mainly around 250–1600 m^2^ g^−1^. On the contrary, M/COF-covered monolith only exhibits 2.5 –40 wt% M/COF loadings and the majority of surface area smaller than 100 m^2^ g^−1^. This is because the 3D skeleton in the M/COF-covered monolith leads to a large dead mass, resulting in low M/COF content and, in turn, a small surface area. However, on the other hand, it can provide strong mechanical stability and robustness. The compressive strength of reported M/COF-covered monolith is all above 2.5 MPa. Due to the different properties, the M/COF-mixed monolith will be a better choice for applications that require high loading of M/COF, such as gas storage. On the other hand, the M/COF-covered monolith is preferably used in fields that demand high mechanical stability, such as bone implants. In this review, the advancements in preserving the microstructure of the M/COFs are discussed separately for the two kinds of monolith.

## 3D-Printed M/COF-Mixed Monoliths

**Fig. 3:**
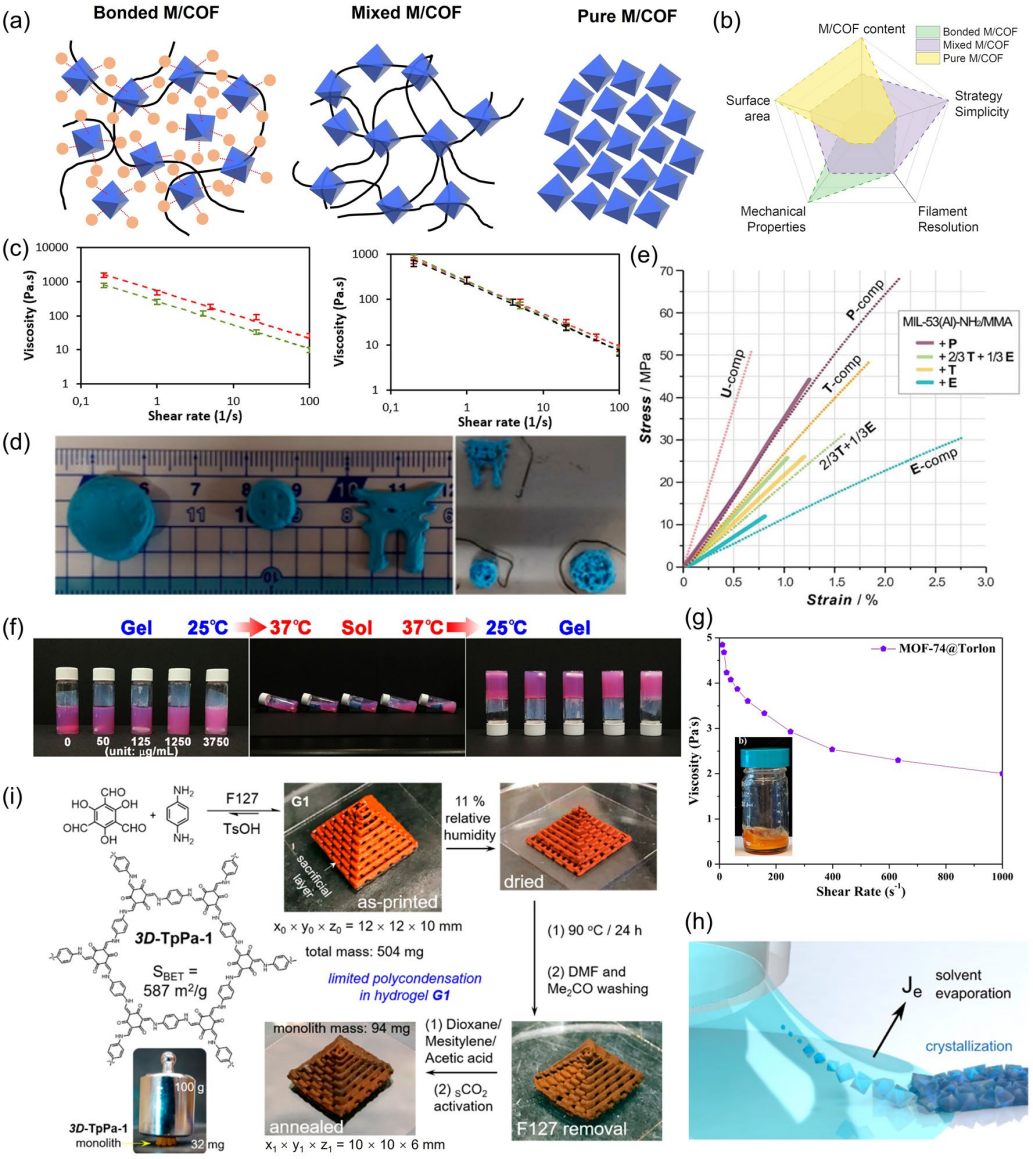
**a** Illustration of three types of M/COF pastes. **b** Comparison of different properties among the three M/COF pastes. **c** Viscosity of pastes formulated with 35 wt% (green) and 51 wt % (red) of CPL-1 MOF (left), and viscosity plots of a pure HEC solution (black), and HKUST-1-based pastes formulated with 11 wt% of large crystals (red) and with 16 wt% of nanocrystals (green) (right). Reprinted from Ref. [13] Copyright 2020, American Chemical Society. **d** Photographs of CPL-1-based monolith printed under the same conditions with pastes formulated with 35 wt% (left) and 51 wt% (right) of CPL-1. Reprinted from Ref. [13] Copyright 2020, American Chemical Society. **e** Stress and strain curves of pure P, E, T, U and 2/3 T + 1/3E 3D-printed materials (dotted lines) and their MOF-based composites (solid lines). Reprinted from Ref. [37] Copyright 2021, Royal Society of Chemistry. **f** Photograph images showing the gel state of PUG-ZIF-8 composite bioinks at 25 °C and the sol state at 37 °C. Reprinted from Ref. [25] Copyright 2021, American Chemical Society. **g** Viscosity plot and the photo of MOF-74@Torlon paste. Reprinted from Ref. [39] Copyright 2020, American Chemical Society. **h** Illustration of meniscus-guided 3D printing of HKUST-1. Reprinted from Ref. [55] Copyright 2022, American Chemical Society. **i** Synthesis route of 3D-TpPa-1 through Pluronic F127-templated coassembly followed by postprinting framework reorganization. Inset: a robust 3D-TpPa-1 cubic lattice loaded with a 100 g weight. Reprinted from Ref. [56] Copyright 2019, American Chemical Society. (Color figure online)

In general, the fabrication of M/COF-mixed monoliths includes three steps: preparation of M/COF-contained pastes, 3D printing, and post-treatment. The simple process makes it much faster to prepare a M/COF-mixed monolith than a M/COF-covered monolith, as the later one requires step-by-step *in situ* growth of M/COF and takes a normal time cost of 13–120 h [34]. A typical paste consists of active materials (M/COFs in this case), additives, and solvents. Based on the composition and interaction between M/COFs and the additives, pastes can be divided into three types: mixed M/COF pastes, pure M/COF pastes, and bonded M/COF pastes, as illustrated in Fig. 3a. For mixed M/COF pastes, the M/COF particles are physically mixed with the additives with weak or no interaction. The additives are only used to adjust the rheological properties without the ability to stabilize or disperse the M/COF particles. Further reducing the amount of additives to zero will lead to a pure M/COF paste. Achieving such a printable paste with 100 wt% M/COF content as well as maintaining the structural integrity of the M/COF monolith requires rather strict control of M/COF rheological properties and necessary additional treatment. For bonded M/COF pastes, the M/COFs either interact with the additives through strong bonds, such as hydrogen bond, or are directly grown on the additive surfaces. This results in higher stability and stronger mechanical properties for the M/COF monolith, but it currently requires a larger amount of additive(s) in the paste. A more detailed comparison between the three pastes is shown in Fig. 3b. In order to develop an advanced paste formula for higher printability, surface area, mechanical properties, and M/COF loadings, all the three components in the paste need to be considered and carefully designed. Besides, different printing methods and post-treatment can also affect the monolith significantly, which have attracted great research interests.

### Advance in Paste Composition

Some general trends on how the intrinsic properties of M/COF affect the 3D monolith have been discovered. The size of M/COFs in the paste has less obvious influence on the viscosity, but a higher amount of M/COFs will cause an increase in viscosity due to the granular feature of the M/COF particles. It has been found that the viscosity increases by more than twofold when the concentration of M/COF is increased by a factor of 1.5 (Fig. 3c). In DIW process, the high viscosity, which causes non-continuous flow, will then lead to a higher pressure during printing and difficulty in replicating the designed model. As shown in Fig. 3a, d discontinuity of extrusion occurs during 3D-printing when the concentration of the MOF increasing from 31 to 51 wt% with a 1.5 fold increases in the viscosity [13]. Moreover, due to the non-adhesive nature of M/COFs, high content of M/COFs will cause a lack of a crosslinked network, which greatly reduces the mechanical strength of the 3D-printed monoliths and makes the printed macrostructure difficult to maintain [31]. The variation in M/COFs types can also cause significant differences in the viscosity. Generally, anisotropic M/COFs, such as 2D M/COFs, are easier to print because during printing, anisotropic materials become oriented in the flow direction under high shear rates, which would minimize the intrinsic viscosity and increases the maximum packing density [13].

The common additives used in the mixed M/COF pastes include organic compounds [13] and ceramic clay [35], such as boehmite, bentonite, PVA, cellulose, F127, and gelatin, while most bonded M/COF pastes only utilize certain polymers, such as cellulose and PA12. This is mainly because the polymer surface has more freedom to be modified for better crosslinking between additives and M/COFs. In general, due to the high flexibility of the polymer, 3D monoliths built using polymer additives normally exhibit large flexural modulus, such as the as-printed 3D ZIF-8/Semiflex [36]. The tensile strength of the 3D-printed M/COF monoliths can also be adjusted through modification on the polymer additives. As shown in Fig. 3e, the 3D-printed MIL-53 monolith with various acrylate-based organic additives delivers different tensile strength [37]. Although the paste with ceramic clay cannot offer high flexibility, its usually has a large compressive strength. In Belmonte et al.’s work, for example, the MOF/boehmite has a compressive strength of around 1 MPa [35]. If changing the boehmite to a mixture of bentonite and PVA, the compressive strength then decreases to 0.48 and 0.56 MPa for the 3D-printed MOF-74(Ni) and UTSA-16 [38]. Normally, the compressive strength can be enhanced by raising the amount of additives. However, a high amount of additives in the monolith will inevitably cause a decrease in the surface area due to their intrinsic low surface area [38–40]. The problem is more serious for polymer additives, which are more likely to cover the surface of M/COFs and block their pores than the ceramic clay, leading to destruction of the intrinsic pore structure [41]. For example, the work using a high PVA content of around 20 wt% can achieve 1.7 MPa compressive strength, but it causes a drop in surface area of around 26% [40]. Besides the difference in mechanical properties, the pastes using polymer additives and ceramic additives also have other distinct properties. Firstly, the amount of polymer additives in a printable paste is normally higher, which leads to a lower content of active M/COFs. The M/COFs content in the paste with single polymer additive is often less than 30 wt%, while by using bentonite ceramic clay, the MOF (ZIF-8) content can reach 66.7 wt% [42]. Secondly, in some cases, such as 3D printing ZIF-7, the PVA binder cannot be used to form a printable paste, but using silica as the additive can lead to a good printability [40]. In addition to pastes with specific degree of viscosity, materials exhibiting sharp *is situ* transition from the fluid to gel state are also commonly used for extrusion. This type of transition is normally based on either chemical reaction between two components or intrinsic physical properties, such as temperature-triggered gelation [22]. M/COFs with additives that has thermal response rheological properties can be easily printed, such as F127, polyurethane-gelatin (PUG), and the thermoplastic binders [25, 43]. The sol–gel transition of PUG with varying temperature is shown in Fig. 3f. However, in order to maintain the transition behavior, the M/COF content cannot be high. For example, the maximum loading of the MOF in ABS is only 10 wt%; otherwise, the filament will become too brittle to maintain the structure integrity [44].

Besides developing pastes with improved printability, higher M/COFs loadings, and enhanced mechanical properties, attention must be given to specific application requirements when designing the paste. For instance, in the biomedical applications, the paste should be biocompatible or biodegradable [25]. Commonly used additives for such applications include polycaprolactone (PCL) [45, 46], PUG [25], poly(lactide-co-glycolide) (PLGA) [47], sericin [28], etc. Although the paste with these additives can be directly printed alone, they often exhibit rather low mechanical strength. In order to form a useable monolith, the M/COF content is generally low for the bioinks, typically less than 10 wt%. The highest recorded content of M/COF in a 3D-printed biocompatible monolith is around 13 wt% using gelatin [48]. Therefore, further improvements are needed to enhance the current M/COF bioink. Additionally, the compatibility of each component should be considered. For example, for the water sensitive M/COF, non-aqueous solvent should be used. In the work by Grande et al., for example, IPA was utilized with hydroxypropyl cellulose and boehmite as the additives to prevent the degradation of UTSA-16 MOF [49]. This, in turn, limits the selection of some conventional components. Hence, the development of a new paste formula would be necessary to achieve an ideal monolith with sufficient mechanical strength, superior printability, high surface area, and suitable for required occasions.

Using mixed additives can be a better option that can result in more advanced properties compared to using the single additive. For example, the inclusion of bentonite binder can enhance the rigidity of 3D-printed structures, while employing an organic binder allows fine-tuning of the rheology of the 3D-printable paste. When using a mixed binder of bentonite and PVA, the loading of MOF can reach as high as 85 wt% [38]. The combination of different polymer additives can also result in better printability with a reduced amount of usage. Compared to the common single-phase organic binders for 3D printing, such as PVA, Pluronic F127, PEO, and polyvinylpyrrolidone K25, a combination of 2-hydroxyethyl cellulose (HEC) and PVA as a mixed-additive was only required for less than 10 wt% to achieve a 3D-printed monolith, due to its high viscosity, which maximizes the porosity [13]. Mixed additives can also enhance mechanical properties. For instance, a combination of various biocompatible polymers, such as PLGA, PVA, PCL and collagen, can improve the mechanical properties of the current bioinks [46]. Besides, in the other work, the additional of EB in the paste has also demonstrated an increase in the storage modulus. The glass transition temperature of the paste with EB is also increased, making the printing process easier [50].

New types of paste compositions have also been developed to solve the problems of unfavorable paste rheology, limited printability, and weak mechanical properties, which common M/COF pastes normally exhibit. One work has demonstrated the use of liquid binder-polyamide(imide) (Torlon), which greatly affects the rheology of the paste by reducing the viscosity and decreasing the required pressure for printing (Fig. 3g) [39]. The additives with dual-functions, which can not only adjust the rheological properties but also help to improve the performance, have also attracts much attentions [51]. 2D Graphene is one example that can be used to facilitate ink extrusion and maintain the printed shape; at the same time, it can increase the electrical conductivity [52]. Other pastes that exhibit a promoted interactions between the components were also explored for improving the mechanical properties of 3D-printed monoliths. For example, graphene oxide has been investigated as an additive, which can form intermolecular hydrogen bonds due to the presence of several donor−acceptor hydrogen-bonding sites (− COOH, − OH, − epoxy) at the edges and on the basal planes. To improve the mechanical properties of 3D-printed monolith using gelatin paste, adding metal ions in the gel paste, such as Ca^2+^, can trigger the crosslinking and work as a filler to enhance the brittleness [48].

To meet the requirement of the de-binding process, additives that can be eliminated without damaging the M/COF are also widely used. If the additive has higher solubility in a certain solution in which M/COF can maintain its structure or a melting temperature lower than the M/COF decomposition temperature, it can be removed in the post-treatment. For example, by treating the 3D-printed ZIF-8/PVDF monolith in hot acetone, PVDF binder can be dissolved with limited destruction on the ZIF-8. The ZIF-8 particles will then appear intertwined through the remaining polymeric web at the surface and are loosely packed in the interior, allowing sufficient exposure. The surface area of the post-treated monolith can reach 706 m^2^ g^−1^ [36]. The use of thermally stable UiO-66 MOF and a commercial polymer binder can also achieve a BET surface area of 633 m^2^ g^−1^ after the removal of binder by heating. However, the de-binding can cause a fall in the mechanical strength as well. After decomposing the binder by heating, the mechanical strength of 3D-printed UiO-66 is reduced from 22.4 to 4.9 MPa [53]. One other example of the COF-GO monolith containing removable PTSA additive also demonstrated that the original hard and heavy monolith became soft and lightweight, once the PTSA is removed [31].

In addition to the post-treatment, adjusting the paste composition to achieve a pure M/COF paste can also reach the goal of eliminating the additives. Developing an additive-free 3D-printed M/COFs without compromising their intrinsic properties eliminates potential blockage or dead mass in the monolith, allowing a perfect combination of the advantages of designed macrostructure and the synthesized microstructure. For example, Wang et al. have developed a facile method to synthesize pure MOF gel based on fast nucleation and slow growth of MOF particles using a high concentration of MOF precursors. The MOF gel paste can be easily extruded and shaped into different architectures due to the strong inter-particle forces that maintain the structure. The as-synthesized MOF gel exhibits a much larger surface area than the gel synthesized by traditional methods, but it still cannot match the surface area of MOF powder [54]. Inspired by the self-crosslinking of M/COFs, the precursors of M/COFs can be made into the paste instead of using the M/COFs themselves and the crosslinking is triggered after 3D printing. Kim et al. demonstrated the use of a liquid ink with 1.1 mM Cu(NO_3_)_2_·3H_2_O and 0.6 mM trimesic acid in 1 mL of dimethyl sulfoxide (Fig. 3h). The solvent quickly evaporates once the filament is extruded from the micro-sized tip, and the MOFs crystallization occurs. The HKUST-1 made using solution-mediated crystallization without the use of additives shows 1192 m^2^ g^−1^ BET surface area, which is superior or comparable to the values obtained using other 3D printing approaches [55]. In another work, Ke et al. mixed the COF precursors with F127 at the initial stage. The imine- and *β*-ketoenamine-based COFs were polymerized in the presence of F127 template. The polymerization degree of the COF is purposely limited to prevent the formation of large COF particles and hindrance of the 3D printing. The amorphous COF is then heated for further crystallization, and the F127 is removed after the 3D printing, resulting in a binder-free 3D COF monolith (Fig. 3i) [56].

### Advance in Printing Methods

In addition to the paste composition, the 3D printing process can also significantly affect the printability, mechanical strength, and texture properties. To maximize the loading and intrinsic properties of M/COFs, considerable efforts have been made to improve the current printing methods in the pre-printing, in-printing, and post-printing process.

**Fig. 4:**
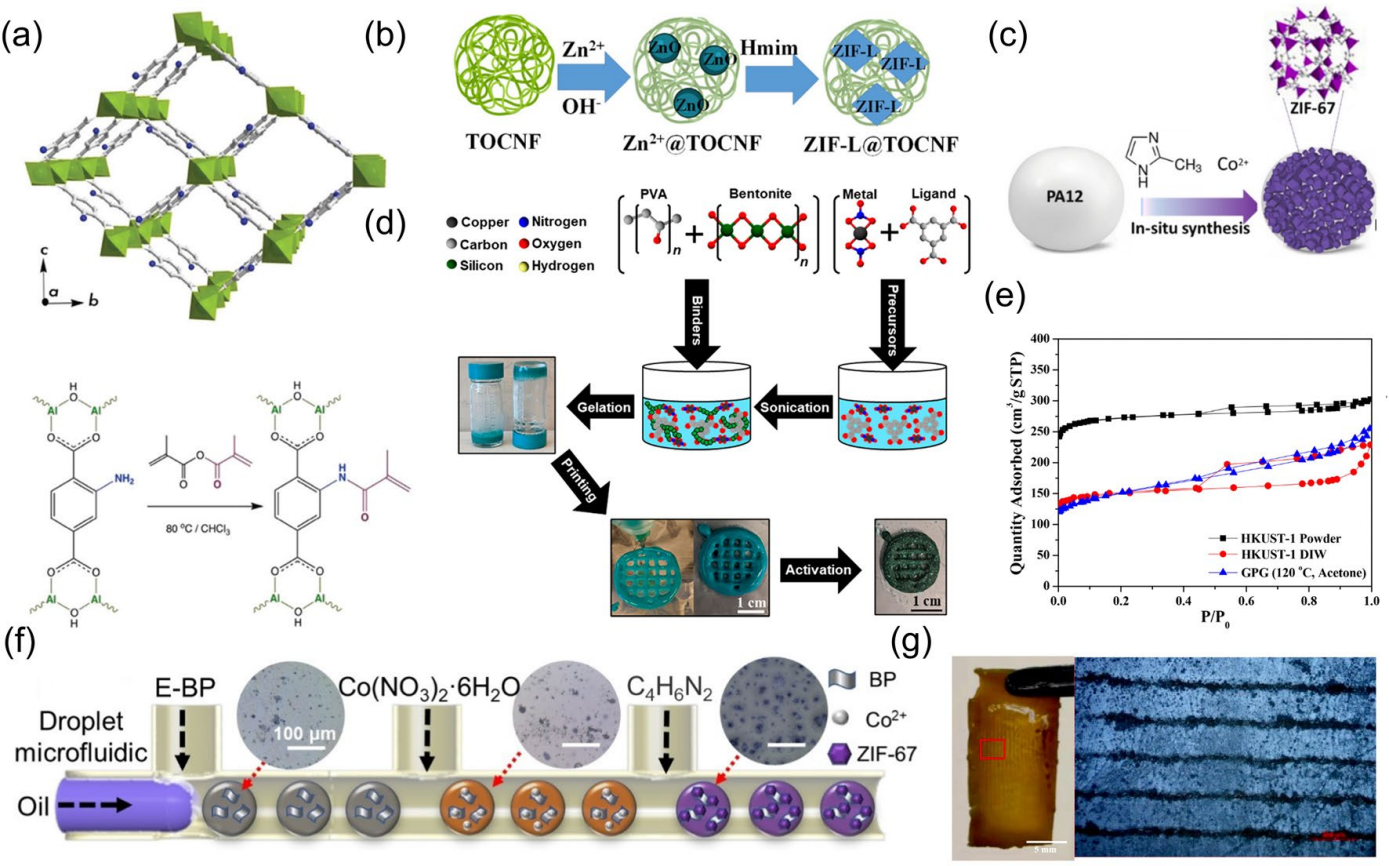
**a** Representation of MIL-53(Al)–NH_2_ structure in the open pore configuration, where AlO_6_ are indicated as green polyhedra, and gray and navy dots are carbon and nitrogen atoms, respectively (top); the schematic of the modification of MIL-53(Al)–NH_2_ structure with methyl methacrylic anhydride (bottom). Reprinted from Ref. [37] Copyright 2021, Royal Society of Chemistry. **b** Schematic for the *in-situ* synthesis of ZIF-L in TOCNF. Reprinted from Ref. [57] Copyright 2023, Royal Society of Chemistry. **c** Schematic of the preparation of ZIF67-PA12 nanocomposite powders. Reprinted from Ref. [59] Copyright 2020, The Authors. Published by Elsevier B.V. **d** Schematic of HKUST-1 monolith formulation by novel GPG technique. Reprinted from Ref. [60] Copyright 2020, American Chemical Society. **e** N_2_ physisorption isotherms for DIW monolith, HKUST-1 powder, and 120 °C-synthesized samples washed in acetone solvents. Reprinted from Ref. [60] Copyright 2020, American Chemical Society. **f** Four-channel droplet microfluidic synthesis of E-BP/ZIF-67 (Inset: optical images of E-BP, E-BP/Co^2+^ and E-BP/ZIF-67 microdroplets). Reprinted from Ref. [51] Copyright 2021, Wiley‐VCH GmbH. **g** Images of MIL-53@ABS film and MIL-53 particle patterns in the MIL-53@ABS film. Reprinted from Ref. [61] Copyright 2020, Elsevier B.V

One major improvement direction in the pre-printing modification is to functionalize the M/COFs or additives to enhance their interaction, which has been well studied for preparing bonded M/COF pastes. Promoting the bonding between M/COFs and additives in the paste will significantly enhance the dispersion of M/COFs, preventing M/COF particles from aggregating together. Several methods have been tried to functionalize the M/COFs to achieve bonding. For example, MIL53(Al)-NH_2_ was functionalized with methacrylic moiety to bond with the commercial photopolymerizable acrylic oligomer and create a 3D printable paste (Fig. 4a) [37]. This strategy can enable a 12 wt% solid content of MOFs in the final 3D-printed monolith. Additionally, additives with specific functional groups can be chosen or synthesized to attract the M/COFs or their precursors for M/COF growth. Carboxylic and hydroxyl groups are well-studied examples of functional groups that can coordinate with the Zn^2+^, ensuring the crystal formation of ZIF-L on the organic compounds (Fig. 4b). The 3D-printed ZIF monolith using CelloZIF-L inks prepared by this method achieved a high ZIF content of 84 wt% [57]. The other work of 3D-printed CelloZIF-8 exhibits a high specific surface area of 900 m^2^ g^−1^ [34]. For SLS 3D printing, since the M/COF is distributed throughout the whole filament, the amount of exposed active M/COF will be limited. Weidner et al. fabricated a MOF-ABS filament for FDM 3D printing. The overall volume fraction of HKUST-1(Cu) is 1%; however, the volume fraction of Cu elements with contact to the outer surface was only 0.036%. This indicates that only a small amount of the HKUST-1 is located on the external polymer surface [58]. Considering this problem, a more advanced modification on the MOF-PA12 paste has been investigated by growing the ZIF-67 on the surface of PA12 particles (Fig. 4c). Although the loading of ZIF-67 in the monolith is only 2.6 wt%, it enables a better dispersion of ZIF-67 crystal and sufficient exposure [59].

During the 3D printing process, the presence of large M/COF particles can lead to favorable shear-thickening behavior, resulting in slower printing speeds. Moreover, certain solvents required for printing can also cause decomposition of some M/COF particles, necessitating lengthy solvothermal growth to regenerate them. To address these issues, the gel-print-grow method has been developed (Fig. 4d) [60]. In this approach, MOF precursors are first prepared into a gel for 3D printing, which helps avoid the problems associated with the large particle size and MOF decomposition in solvent. The MOFs can then be formed in situ by applying heat, removing the solvent, and activating the MOFs, which simplifies the optimization of printing parameter and benefits for universal 3D printing of M/COFs. The resulting activated monolith achieved 83% of the powder’s N_2_ adsorption capacity (Fig. 4e). Similar insights have been applied in microfluidic 3D printing (Fig. 4f) [51]. For example, in the case of black phosphorus (BP), cobalt source, and 2-MIM, added into a microfluidic device, the MOFs can be rapidly formed on the BP during printing, enabling a smooth printing process. Furthermore, strategies to control the exposure of M/COF to increase the active area have been investigated. Wang et al. applied an acoustic field to a liquid photosensitive paste containing MIL-53(Fe). The MIL-53 was patterned into parallel lines, and the stereolithography was then used to prepare a polymer film in situ. This method allowed for better control of the dispersion of MOF particles in the polymer film (Fig. 4g) [61].

Process parameters also play a crucial role, especially in SLS 3D printing. The layer formed by the laser consists of a densely sintered top portion and a partially sintered porous bottom portion. It would be essential to strike a balance in laser power to avoid degradation of the polymer, leading to reduced mechanical strength, or insufficient power, resulting in a weakly sintered film. Therefore, carefully reducing the sintering power and time to achieve minimally sintered top portion with more voids while maintaining sufficient mechanical strength is ideal [62]. By optimizing the printing conditions in SLS, it leads to the formation of a solid, porous, powder-bed-like object, where the sintered polymer particles retain their particle-like appearance and contain accessible voids between them. The functional additive mixed with the printing matrix is attached only on the surface of the sintered particles, allowing for interactions with the fluid passing through the material [15].

**Fig. 5:**
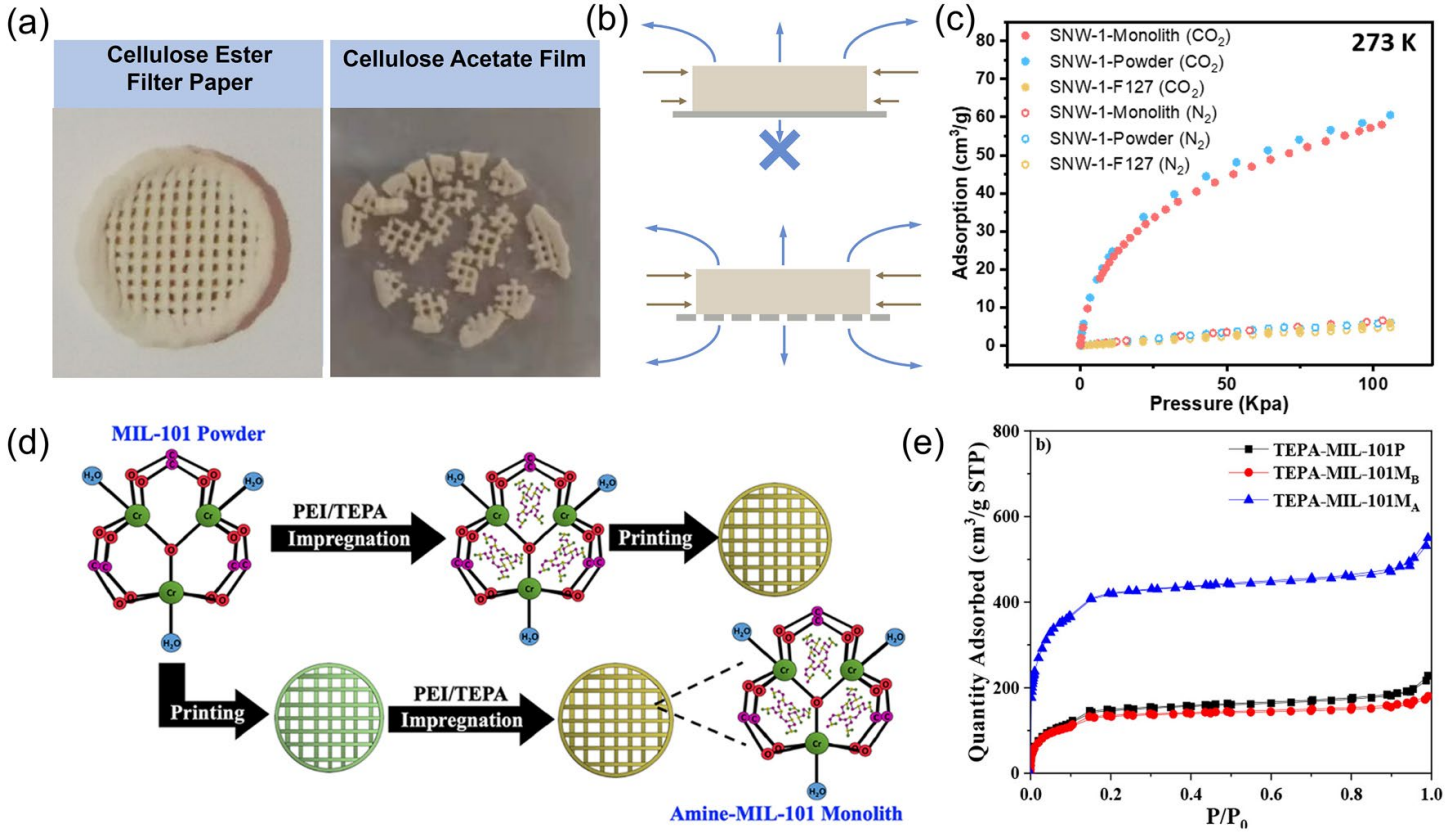
**a** Appearance of binder-free 3D printed COF using different drying substrate. Reprinted from Ref. [63] Copyright 2020 Elsevier B.V. **b** Schematic of the drying mechanism of 3D-printed monoliths on porous and non-porous substrates. Reprinted from Ref. [63] Copyright 2020, Elsevier B.V. **c** CO_2_ and N_2_ adsorption curve of SNW-1 monolith, SNW-1 powder, and SNW-1/F127 monolith at 273 K. Reprinted from Ref. [63] Copyright 2020, Elsevier B.V. **d** Formation processes for pre- and post-impregnated MIL-101 monoliths. Reprinted from Ref. [64] Copyright 2019, American Chemical Society. **e** N_2_ physisorption isotherms for TEPA-MIL-101 powder, 3D monolith with pre-penetrated MOF, and 3D monolith with post-treatment. Reprinted from Ref. [64] Copyright 2019, American Chemical Society

Controlling the post-treatment is also important for enhancing the properties of 3D-printed M/COF monoliths. Indeed, the drying process is a critical step in the post-treatment, and it has been found that slow drying at lower temperatures, allowing for shrinkage during drying, leads to a denser crystal packing and higher mechanical strength [13]. However, a nonuniform shrinkage rate in the monolith can result in cracks and compromise structure integrity. To address this issue, Wang et al. has utilized a porous drying substrate to enable a uniform drying speed (Fig. 5a, b). This approach reduces the build-up of stress in the 3D printing monolith during the drying process, which significantly helps to maintain the structural integrity of the 3D-printed monolith with even no additives in the paste. As a result, the as-fabricated additive-free 3D monolith shows almost similar surface area, pore size distribution, and gas separation performance as the initial powder sample (Fig. 5c) [63]. Post-treatment can also be used to modify the M/COFs after shaping them into a 3D monolith. One example is post-impregnation, which introduces guest molecules into the M/COF after 3D printing. For example, Rezaei et al. impregnated TEPA and PEI into MIL-101 by immersing the 3D-printed monolith in the corresponding solution (Fig. 5d). The post-impregnated monoliths exhibited a fourfold increase in surface area and pore volume compared to their pre-impregnated counterparts, which could well be attributed to the reduced amine-pore diffusion (Fig. 5e) [64].

## 3D-Printed M/COFs-Covered Monolith

The 3D M/COF-covered monolith is often chosen when a mechanically strong support is the primary requirement. In applications that demand a stable scaffold, such as bone repair, the 3D-printed M/COFs-covered monolith proves to be a better choice [65, 66]. M/COFs-covered monolith is achieved by directly growing or coating M/COFs on the surface of the 3D-printed monolith. The M/COFs deposition need to be controlled to minimize any adverse effects on its mechanical performance. This types of monolith effectively addresses several issues that may arise in the M/COF-mixed monolith, such as M/COFs embedded in the polymer [67], M/COFs-covered by the additives [68], lack of controllability and flexibility [69], poor particle matrix junction [70], and low mechanical strength [71]. Moreover, the stable monolith can be re-used and the ineffective M/COFs coating after long-time usage can be removed and regenerated by growing the M/COFs layer again, which significantly reduces the cost [72, 73]. However, there are still some challenges that need to be addressed, including low M/COFs loading and nonuniform deposition. The recent research and optimization to overcome these challenges and fully exploit the potential of the 3D M/COFs-covered monolith are introduced in this section.

### Advance in Scaffolds

Various materials can be used to fabricate the 3D scaffolds, each tailored to meet certain specific requirements, such as the high flexibility, biocompatibility, high stiffness, or high electrical conductivity [74–76]. However, caution is needed when selecting the materials, as the M/COF precursor solution may cause the degradation of scaffolds under certain conditions, such as in a too acidic environment or due to high water solubility of the scaffolds, resulting in a reduction of mechanical strength [75]. In the work by Zhang et al., for example, silica was chosen as the skeleton material due to its resistance to degradation in organic solvents and at high temperature, which makes it a suitable alternative to ABS or PLA [77]. Similarly, the use of kaolin-based support instead of silica and bentonite clay was preferred, as the latter materials are prone to disintegration upon exposure to the synthesis liquor [78]. By carefully selecting compatible materials for 3D scaffolds, one can ensure the stability and mechanical integrity of the final monolith.

To enhance the M/COFs loading and create more surface area in the 3D skeleton, etched skeletons have been widely employed. For example, Wang et al. etched the skeleton in the KOH solution to remove the SiO_2_ nanoparticles on the surface and generated more pores in 3D SiO_2_ skeleton [79]. Similar approaches have also been taken, such as removing the PLA polymer using DMF or ABS using acetone for 3D PLA scaffold [67, 80]. In addition to etching the skeleton to create more growing sites, modifying the skeleton surface to provide stronger adsorption and improve the M/COFs growth is also effective to enhance the M/COFs loading. Polymer supports are particularly advantageous for improving M/COF growing sites, as their surface can be easily modified and functionalized by various methods [81]. For example, the existing carbonyl group and carboxyl group in PLA can efficiently bind with Cu atoms in Cu-MOFs through the coordination process, producing Cu-MOFs/PLA films [73]. Another approach involved forming a polydopamine (PDA) layer on a etched 3D SiO_2_ skeleton to facilitate the encapsulation of MOF particles within the hierarchical structure [79]. These methods effectively provide more surface area on the skeleton and enhance the M/COFs loading, thereby optimizing the performance of the 3D-printed M/COF monoliths.

### Advance in Covering Methods

**Fig. 6:**
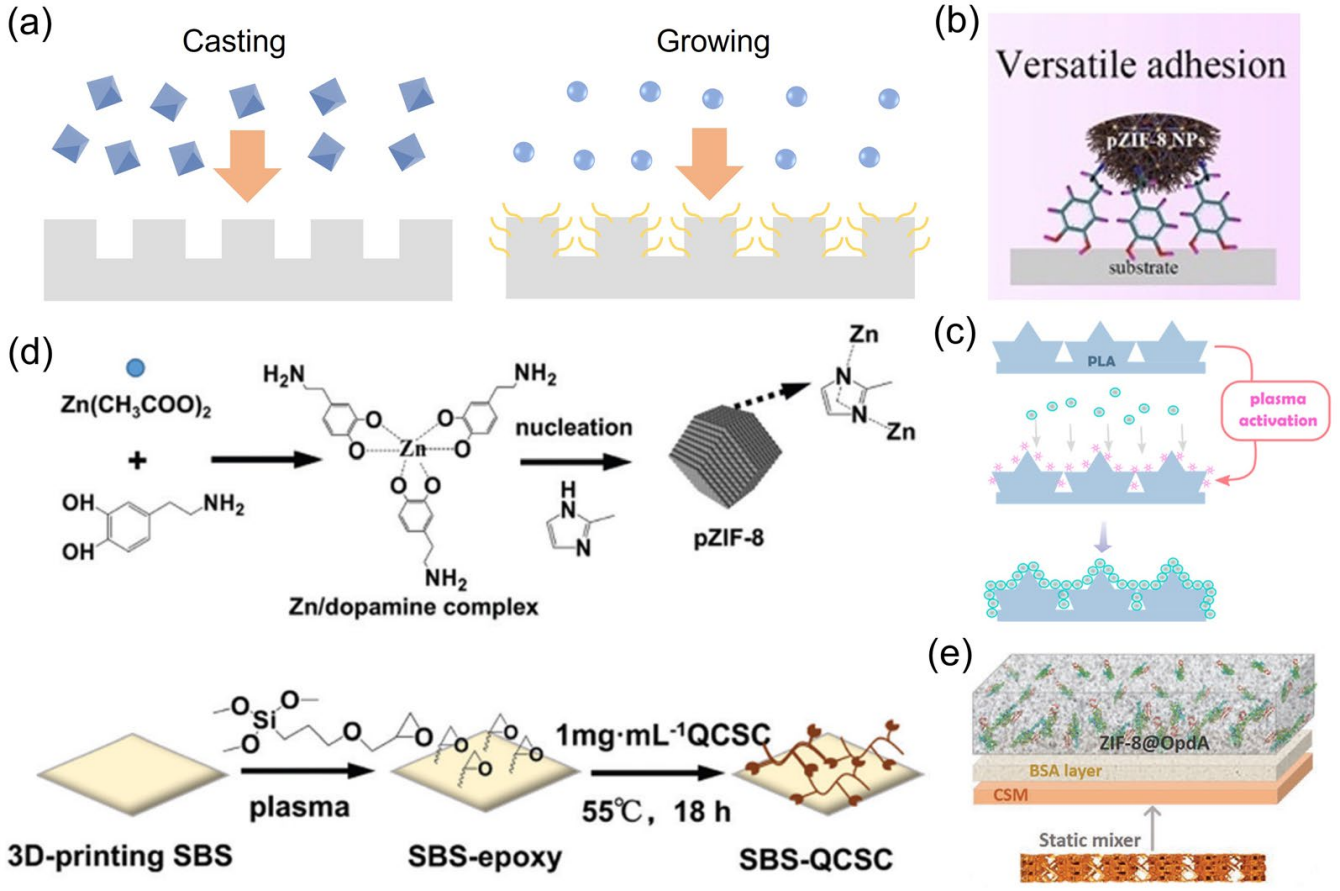
**a** Schematic of the two different M/COF covering methods. **b** Schematic of the pZIF-8 nanoMOFs attaching to the substrate. Reprinted from Ref. [87] Copyright 2021, Elsevier Ltd. **c** Strategies employed to immobilize active nanoparticles on the plasma-treated PLA support. Reprinted from Ref. [81] Copyright 2020, American Chemical Society. **d** Schematic of the preparation of pZIF-8 and SBS-QCSC substrate. Reprinted from Ref. [88] Copyright 2022, Royal Society of Chemistry. **e** *in situ* MOF growth and encapsulation process. Reprinted from Ref. [92] Copyright 2020, WILEY‐VCH Verlag GmbH & Co. KGaA, Weinheim

A number of research works are devoted to developing advanced M/COFs deposition methods for 3D M/COFs-covered monolith. These methods can be categorized into two types: directly coating M/COF particles and growing M/COF particles, as shown in Fig. 6a. In the direct coating approach, M/COF particles are synthesized and then loaded onto the surface of the 3D-printed scaffold separately; while the growing method entails immersing the 3D monolith in a precursor solution as well as allowing M/COFs to nucleate and grow on the surface of the scaffold. Both of these methods offer unique advantages and have been explored extensively to achieve more advanced 3D-printed M/COF monoliths.

The direct coating approach offers a simple and straightforward method for creating 3D-printed M/COFs-covered monolith. In the traditional preparation process, a M/COFs dispersion is used to create a M/COFs ink, which is similar to but much less viscous than the ink used in the preparation of 3D-printed M/COFs-mixed monoliths. The M/COFs dispersion is then either dipped onto the 3D lattice [82, 83] or the lattice is immersed in the M/COFs dispersion to produce the 3D-printed M/COF-covered monolith [84, 85]. The adhesion between the M/COF and the skeleton is achieved by using the additives, such as Chitosan [84, 86], and PVDF [82]. However, a major challenge is the potential weak adhesion between the M/COFs and the monolith, which can lead to poor stability in applications. To improve adhesion, Lu et al. have developed a PDA-hybridized nano-sized ZIF-8 (Fig. 6b). The catechol motifs on the ZIF-8 surface enable versatile adhesiveness, which is similar to the strong adhesion between mussels and various surfaces due to the abundant amount of L-DOPA contained proteins [87]. Furthermore, surface modification of the 3D skeleton can be employed to attract M/COF more strongly. Jin et al. for example, have developed a plasma activated 3D-printed skeleton, where the surface is functionalized with hydroxyl or hydroperoxyl groups (Fig. 6c). The modified Fe-MOF with PAA shell can then be bonded to the surface through coordination bonds, significantly enhancing the loading and dispersion of Fe-MOF [81]. In another study, a combination of plasma-treated 3D-printed SBS and mussel-inspired polydopamine-decorated ZIF-8 was used (Fig. 6d). This resulted in firm deposition of ZIF-8 on the SBS surface [88]. These innovative approaches are promising for achieving strong and stable M/COFs coverage on 3D-printed monoliths.

Numerous works have also demonstrated the immersion of 3D skeleton in M/COF precursor solutions for growing M/COFs on their surfaces, such as Zn/Co-MOF@TCP [75], ZIF-L MOF@PA [89], Co-,Cu-MOF@SiO_2_ [68, 77], and PCN-224 MOF@Ca_2_SiO_4_ [74]. By repeatedly immersing a 3D monolith in the precursor solutions, the M/COF loading can be controlled [73, 90]. In addition, the concentration of the precursor can also affect the loading of the M/COF [91]. However, it should be noted that secondary growth of M/COFs on the 3D-printed M/COFs monolith can lead to a significant drop in compressive strength, imposing a limitation on the M/COFs growth [39]. To facilitate the uniform growth and achieve higher loading, the skeleton should provide sufficient nucleation sites. Some materials, like the alginate matrix, can effectively coordinate the ligand, facilitating M/COFs growth on their surface [69]. For instance, one work has prepared a mixed surface by dip-coating the 3D skeleton in a bovine serum albumin (BSA) solution. The BSA forms a bio-surface which provides both anchoring and nucleation sites to initiate ZIF-8 growth (Fig. 6e) [92].

**Fig. 7:**
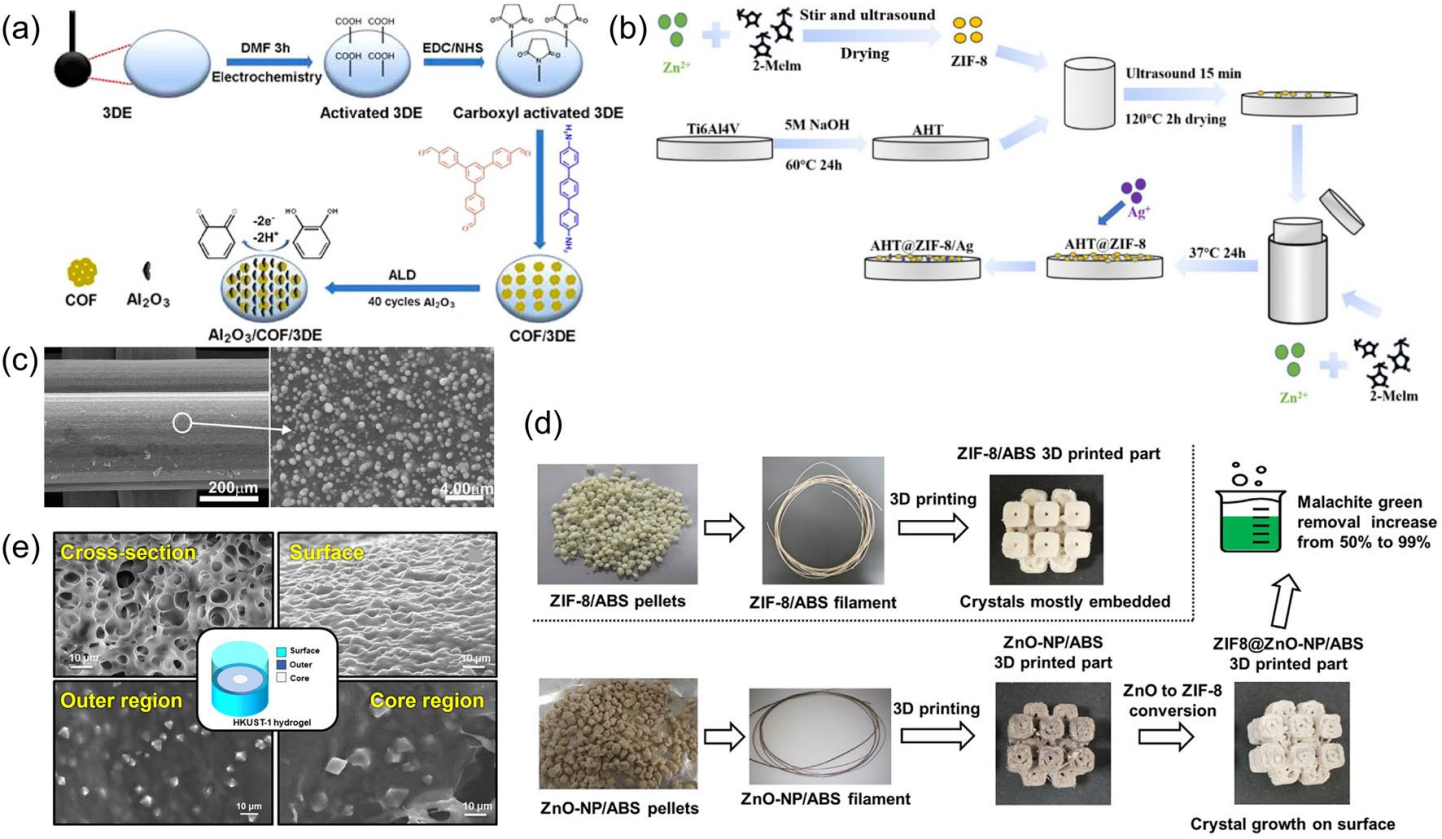
**a** Schematic of the construction of the COF on 3D-printed COOH-modified nanocarbon electrodes. Reprinted from Ref. [93] Copyright 2022, American Chemical Society. **b** Preparing of ZIF 8 and ZIF 8-Ag coating on Ti_6_AlV_4_ sheet. Reprinted from Ref. [94] Copyright 2022, Jilin University. **c** SEM observation of an ABS filter coated with ZnO. Reprinted from Ref. [71] Copyright 2020, The Korean Society of Industrial and Engineering Chemistry. **d** Schematic of the preparation of ZIF-8 and ZnO-NP composite filaments for FDM 3D printing; the monolith obtained by conversion of ZnO to ZIF-8 shows better malachite green removal ability. Reprinted from Ref. [72] Copyright 2021, Elsevier Ltd. **e** SEM images of the 3D-printed hydrogel samples; the HKUST-1 is grown both at the outer and core regions. Reprinted from Ref. [70] Copyright 2020, American Chemical Society

For skeletons with inert surfaces that do not readily facilitate M/COFs growth, modifications are necessary. For instance, one work electro-oxidized the 3D-printed carbon to introduce COOH functional groups on the surface for growing COFs. This step provides activated carboxyl groups that enable functional COF materials with numerous amino groups to cross-link on the monolith surface through covalent bonding (Fig. 7a) [93]. In some other cases, a small amount of M/COFs can also be deposited as seeds on the surface to facilitate the subsequent M/COFs growth. For example, 3D-printed Ti_6_AlV_4_ was sonicated in a 1 wt% ZIF-8 solution and then immersed in the ZIF-8 precursor solution for hydrothermal synthesis to achieve a rather uniform ZIF-8 coating (Fig. 7b) [94]. Sometimes, it can be very challenging to form a uniform seeding on complex 3D structures. To tackle this problem, advanced deposition methods, such as atomic layer deposition (ALD) can be used to ensure a uniform distribution of M/COF on the surface of skeletons. The ALD-deposited ZnO on ABS, for example, exhibits a uniform distribution (Fig. 7c). After conversion, homogenous and continuous ZIF-8 crystal layers are present both on the outer walls and inner areas of the 3D-printed monolith [71].

Another approach involves mixing one of the M/COF precursors in the paste first and then reacting it with the other precursors to form M/COFs by immersing the 3D-printed monolith into the solution containing the other precursors. This method allows the growth step to occur in the inner part of the monolith, increasing the effective M/COFs loading and achieving more homogeneous composites. For instance, Ca-contained or Co-contained monolith 3D-printed monolith can be immersed in a linker solution [67, 78], and the linker-containing 3D monolith can be immersed in lanthanide-containing solution [69]. In addition, researchers have also explored the incorporation of metal oxide as metal precursors in the paste. For example, ZnO-ABS filament was fabricated, and the MOFs crystal growth was conducted during the post-printing treatment. This approach has been proven to offer higher performance compared to directly making Zn-MOF filament for 3D printing Zn-MOFs (Fig. 7d) [72]. In the case of an *in-situ* grown HKUST-1 monolith using this method, the MOFs was observed to grow all over the 3D-printed skeleton and even within its interior (Fig. 7e). The HKUST-1 crystal exhibits a higher density and smaller size (approximately 5.5 μm) near the surface, while the crystals were bigger (around 10 μm) toward the interior. This difference in density and size distribution is likely due to variations in the ligand concentration and distribution of Cu^2+^ ions, as well as the limitations imposed by diffusion-limited growth in the bulk [70]. This represents an advantage over M/COFs-polymer composites prepared through direct blending methods or surface in-situ growth techniques.

## Conclusion and Future Perspectives

3D printing technology brings plenty of advantages and opportunities to the MOF and COF materials; however, it also requires more efforts on the preparation of suitable M/COF pastes and development of the new 3D printing methods. In this review, the status of the 3D printed M/COFs has been introduced. It has been found that the current 3D-printed M/COFs can be divided into the 3D mixed monolith and 3D covered monolith, which have distinct properties and often applied in different areas. The recent advances in design strategies regarding to both the paste/scaffold formation and the 3D-printing process for achieving better structural features (surface area, porosity, and micromorphology) for the two types of 3D printed M/COFs are also illustrated.

The State-of-the-arts 3D printed M/COFs are illustrated in Table 1. The currently known strategies have shown promising results in achieving M/COFs-mixed monolith with loadings of higher than 80 wt%, compressive strength around 1 MPa, and approximately 70% surface area compared to their powder counterparts. Among all the reported data, the 3D-printed UTSA-16(Co) using bentonite and PVA as binders shows the highest MOF loadings of 85 wt%, and the 3D printed ZIF-8-ZnO@TOCNF presents the largest surface area, but they exhibit lower compressive strength. The 3D printed MOF-74 with Torlon as the additives shows the highest compressive strength of 637 MPa, while the surface area and loadings are quite small. Further increasing the M/COFs loadings while maintaining the required mechanical properties and printability is challenging with the use of mixed M/COF pastes. The physical mixture approach may not be able to ensure an optimal dispersion, and a certain amount of additive is often required to maintain an ideal range of rheological properties. There are certain reported pure 3D printed MOFs and COFs, but the mechanical properties are either not reported or very small. While the goal of achieving a pure M/COF paste is desirable in the future, the currently faced limitations of low mechanical strength and limited applicability make it difficult to implement in the near term. On the other hand, the M/COFs-covered monoliths typically have M/COF loadings below 30 wt% and surface areas of around 10 m^2^ g^−1^. However, there has been a notable progress in the fabrication of MOF@kaolin monoliths, which demonstrated a high M/COF loading of 90 wt%, compressive strength of 4.75 MPa, and a remarkable 98.4% of the powder surface area. The success of this monolith is attributed to the MOFs not only covering the entire surface, but also growing into the interior of the filament in the 3D monolith. The challenge with the fabrication process of 3D M/COFs-covered monolith is that it is more complex compared to the 3D M/COFs-mixed monoliths. The investigation into both types of 3D M/COFs monoliths suggests a common ideal 3D-printed M/COFs structure with fully covered surfaces, maximized M/COFs dispersion in the interior, a connected light matrix, and strong interactions between the M/COFs and the matrix.

**Table 1:** State-of-the-arts 3D printed M/COFs

Materials	Loadings (wt%)	Surface area (m^2^ g^−1^)	Compressive strength (MPa)	Type
3D-printed UTSA-16(Co) [38]	85	568 (Powder: 727)	0.56	Mixed monolith
3DP ZIF-8-ZnO@TOCNF [34]	70	2330	NA	
3DP MOF-74/Torlon [39]	13	80 (Powder: 1180)	637	
3DP HKUST-1 [55]	100	1192	NA	
Mg-MOF 74@3DP Ti [85]	NA	NA	71.42	Covered monolith
UTSA-16@3DP Kaolin [78]	90	620	4.75	

**Fig. 8:**
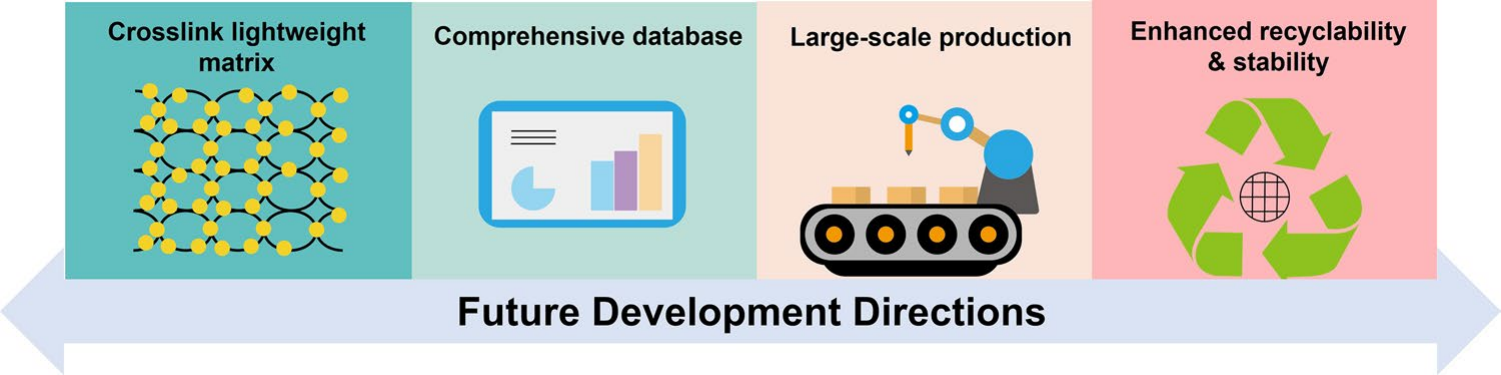
Proposed future development directions

In future studies on 3D-printed M/COF monoliths, four aspects as shown in Fig. 8 are recommended for further investigation: (i) exploring the use of lightweight additives which can crosslink with the M/COFs particles to generate a mixed matrix. Achieving such lightweight additives would allow for large loadings, high dispersion, and excellent exposure of the MOFs and COFs, while also providing sufficient mechanical strength through the M/COFs-additive matrix. By optimizing the paste formulation with bonded M/COFs, one can work toward obtaining the ideal combination of properties for various applications. (ii) Reporting a comprehensive set of data, including the mechanical strength, M/COFs loadings, surface area, and pore structures for both the monoliths and powder counterparts. Such detailed information will enable researchers to understand the effectiveness of different 3D printing methods, ensure a better comparison across different studies, and identify new areas for further improvement. (iii) Developing a general and facile fabrication approach that allows for large-scale production of 3D-printed monoliths using different M/COFs materials. A standardized and easy-to-use method would facilitate the widespread adoption of this technology and open up possibilities for various new applications. (iv) Enhancing the stability and recyclability of the 3D-printed M/COFs. Since the 3D printed monoliths might be used in different harsh environments, it is important to investigate methods to increase their stability and recyclability, which will be valuable for sustainable applications. With the continuing efforts, there will be great opportunity for 3D-printed M/COF monoliths with preserved microstructure and intrinsic properties to be successfully developed. This, in turn, will enable the development of more advanced performance and devices with 3D-printed metal/covalent organic frameworks.
